# Reversal of primary obesity surgery endolumenal following postoperative complications: A case report

**DOI:** 10.1016/j.ijscr.2022.107483

**Published:** 2022-08-11

**Authors:** Khalil Terro, Mohanad Baroudi, Ahmed Abunimer, Saleha Abdul Rab, Areez Shafqat, Belal Nedal Sabbah

**Affiliations:** a Department of Surgery, Specialized Medical Center Hospital, Riyadh, Saudi Arabia; b College of Medicine, Alfaisal University, Riyadh, Saudi Arabia

**Keywords:** Obesity, Endoscope, Primary obesity surgery endolumenal, Bariatric surgery, Endoscopic sleeve gastroplasty

## Abstract

**Introduction and importance:**

Although bariatric surgeries are an increasingly popular option to achieve significant weight loss in patients who fail to do so via conservative measures, these procedures are invasive and carry a risk of complications, many of which are serious and potentially fatal. In this context, endoscopic bariatric therapies (EBT) such as primary obesity surgery endolumenal (POSE) have been proposed as a new minimally invasive weight loss procedure to reduce the risk of postoperative complications. However, these procedures are in their early stages, with only a few cases reported in literature. We report our experience in managing a complicated case of POSE gastroplasty.

**Case presentation:**

We report a case of a 45-year-old woman presenting with severe epigastric pain and vomiting. She was suffering from postoperative complications following a transoral gastroplasty procedure. The patient was managed surgically to correct the offending complication, which consequently resulted in reversal of POSE.

**Clinical discussion:**

Conventional bariatric surgical procedures are generally performed using an open and/or laparoscopic approach. Although highly effective, this approach is associated with significant complications. EBTs are gaining traction as novel treatment modalities for obesity. A major reason for adopting this approach is the fact that endoluminal therapy represents a minimally invasive treatment option for obesity with a minimal rate of complications.

**Conclusion:**

Due to the fact that POSE reversal has never been reported or discussed, especially in the context of gastric perforation, it is imperative that future studies are conducted on the matter.

## Highlights

• Endoscopic bariatric therapies (EBT) like Primary Obesity Surgery Endolumenal (POSE) offer an outpatient and minimally invasive alternative to traditional bariatric surgery.
• Typical complications of POSE are minor and include nausea, vomiting, and abdominal pain, while the rarer, major complications include GI bleeding, perforation, severe pain, or hepatic abscess.
• Conservative management remains the mainstay of treatment for most complications associated with POSE.
• While POSE has been deemed relatively safe, severe complications such as acute abdomen secondary to gastric perforation necessitate revision and reversal of the procedure.

## 1 Introduction

Although lifestyle modifications – including dietary changes and exercise – are the preferred methods of losing weight, they often fail to cause significant long-term weight loss. To overcome these limitations, bariatric surgeries have been performed in subsets of obese patients. However, bariatric surgeries, due to their invasive nature, carry a risk of complications, some of which are serious and potentially fatal [1]. For this reason, a transoral endoscopic technique has begun to attract the attention of many surgeons to avoid such complications. Procedures that use the endoscopic technique, including those such as primary obesity surgery endolumenal (POSE), have been claimed to have minimal and transient postoperative complications due to their minimally invasive nature [2]. These procedures are in their early stages, and the number of cases reported in the literature is limited. Hence, there is insufficient data regarding their short- and long-term complications. Herein, we report our experience in managing a patient with postoperative complications following a POSE procedure. This case report is reported in line with the SCARE criteria [3].

## 2 Case presentation

A 45-year-old woman presented to our clinic with severe epigastric pain and vomiting. Her past surgical history was significant for a primary endolumenal obesity surgery (POSE) one month ago. The patient's past medical history included polycystic ovarian syndrome, hypertension, and recurrent miscarriages. She was admitted for further evaluation and management. The patient was febrile on admission, and laboratory investigations showed leukocytosis and elevated C-reactive protein (CRP). Her Body Mass Index (BMI) was 28.4, blood pressure 117/81, heart rate 107, and respiratory rate 17. She was started on Meronem 2 g IV Q8hrs for 11 days, and IV Anidulafungin 200 mg with on the first day followed by 100 mg once daily for 5 days. She was also given Fentanyl 50Mcg IV four times daily, Diclofenac 1 suppository per rectal twice daily, Ibuprofen 400 mg IV Q8hrs for 3 days, Oxycodone 2 mg/h IV infusion + 2 mg IV bolus, along with Dormicum 2 mg and Ketamine 2 mg IV STAT for severe pain.

**Fig. 1:**
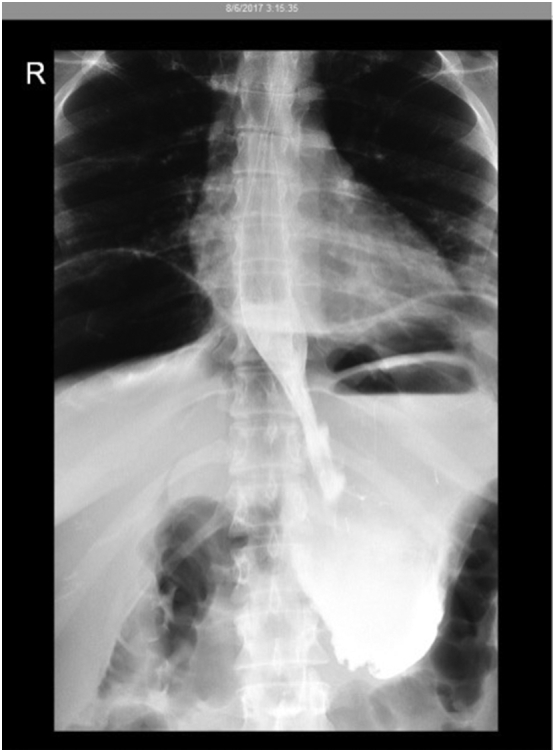
Gastrografin swallow revealing marked free air under the diaphragm, with no definite contrast leak seen. Mild mucosal thickening is suspected at the gastric pylorus. The visualized parts of the distal esophagus and the stomach appear unremarkable.

**Fig. 2:**
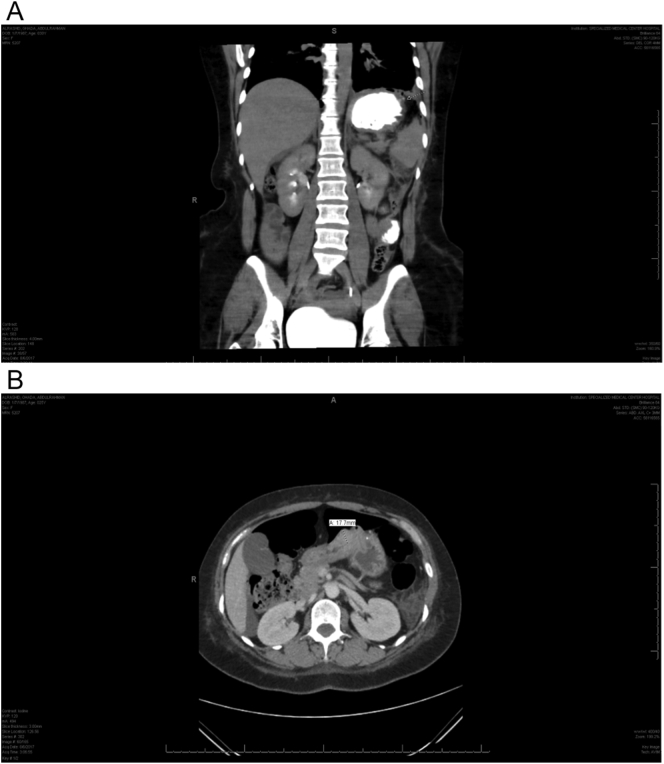
a/b: Abdominal CT with IV and oral contrast showing evidence of a previous surgical procedure along the distal body of the stomach and antrum, with thickening of the antrum wall, and a mildly distended stomach (likely related to the patient's surgical history). The scan also suggests leakage of a track of contrast from the gastric fundus. A large amount of pneumoperitoneum is also seen with intra-abdominal free fluid. Additionally, there is minimal left-sided pleural effusion, with atelectasis of the basal segments of both lower lobes. There is also a small right pleural air bubble, likely caused by air leakage from the abdomen.

A gastrografin swallow was performed by the interventional radiologist on the first day of admission and revealed marked free air under the diaphragm, but with no definite contrast leak (Fig. 1). However, there was suspicion of mucosal thickening in the gastric pylorus. The visualized parts of the distal esophagus and stomach appeared unremarkable. An abdominal CT scan with IV and limited oral contrast was also performed and revealed evidence of a previous surgical procedure along the distal body of the stomach, with thickening of the antrum wall, and a mildly distended stomach likely related to the patient's recent endoscopic procedure. More concerningly however, the CT scan showed leakage of contrast from the gastric fundus, along with a large amount of pneumoperitoneum and free fluid in the abdomen. These findings, when correlated with the patient's clinical findings and results of the gastrografin study, were found to be consistent with gastric perforation. There were also minimal, small, left-sided pleural effusions with atelectasis of the basal segments of both lower lobes, with a small right-sided pleural air bubble likely representing leaked air from the abdomen (Fig. 2). A pigtail catheter was inserted into the left pleura of the patient, which drained 315 cm^3^ of yellowish exudative fluid. A nasogastric tube was also inserted and drained 560 mL of gastric fluids. The patient was kept in the hospital and closely monitored. Pleural fluid analysis revealed normal values. Pleural fluid culture was negative. For the next 6 days, the patient was vitally stable and afebrile but still complaining of mild abdominal pain and left chest pain.

Seven days later, the patient developed severe abdominal pain, vomiting, tachycardia, and elevated temperature. This prompted a diagnostic laparoscopy, which showed a severely dilated proximal stomach secondary to extreme narrowing at the incisura angularis. This narrowing was caused by fragments of Prolene sutures in the gastric body, which was done to the patient when she underwent the POSE procedure. All sutures were pulled out and cut with scissors, which relieved the obstruction, and thereby reversed the POSE procedure. An air-leak test was performed to assess the staple line for obvious leakage, and no bubbling of air was seen. An upper endoscopy was also performed intraoperatively, which revealed no obstruction.

**Fig. 3:**
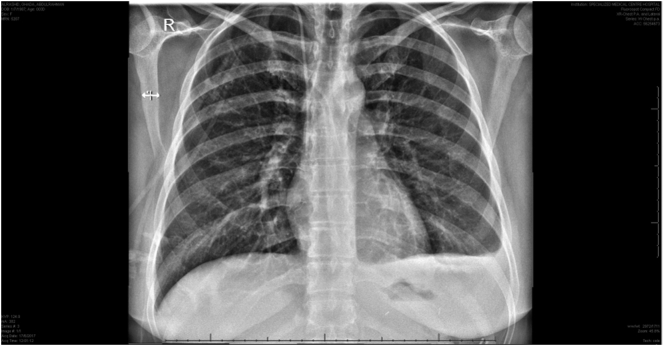
Follow up chest X-ray performed two days after POSE reversal, revealing absence of pneumoperitoneum, decreased left-sided pleural effusion (though still present), and clear lungs.

A chest X-ray performed two days after the surgery showed that the left pleural effusion had decreased, and that there was no pneumoperitoneum (Fig. 3). During her stay at hospital, the patient underwent several sessions of chest physiotherapy and was prescribed analgesics. She was discharged on the 4th day post-operation, with no and further complaints and complications.

## 3 Discussion

The long-term weight loss outcomes of diet, exercise, and weight-reducing medical therapy are relatively poor compared to bariatric surgical procedures [4]. Conventional bariatric surgery is generally performed using an open and/or laparoscopic approach and can be divided into three types: restrictive (e.g., gastroplasty, gastric banding, sleeve gastrectomy), malabsorptive (e.g., biliopancreatic diversion, duodenal switch), or a combination of both (e.g., gastric bypass) [5], [6]. Although highly effective, bariatric surgery is associated with significant complications, with one study determining that severe postoperative complications (including embolism, thrombosis, leakage, abscess, and wound infections) occurred in up to 13 % of patients [1].

Endoscopic bariatric therapies (EBT), such as primary obesity surgery endolumenal (POSE) and endoscopic sleeve gastroplasty (ESG), are gaining traction as novel treatment modalities for obesity. A major reason for adopting these approaches is the fact that endoscopic therapies present a promising alternative to bariatric surgery that is outpatient, minimally invasive, incisionless, has a shorter recovery time, and a much lower complication rate [2]. POSE is an endoscopic procedure that relies on an Incisionless Operating Platform™ (IOP; USGI Medical, San Clemente, CA, USA); it involves placement of interrupted, transmural plications (or folds) in the fundus and preantral area of the stomach using specialized suture anchors [7], [8]. POSE is not to be confused with ESG, another endoscopic procedure that involves placing a running suture pattern that connects the front and back walls of the stomach, bringing the walls together, hence approximating a vertical gastroplasty [9], [10]. It is worth noting that these procedures are relatively new, with the earliest formal presentation of outcome data regarding POSE dating back to 2013 [11].

While it has been established that ESG bears low risk of short-term complications (long-term complications are yet to be assessed) [12], [13] lesser data exists on the complications of POSE. Espinós et al., who operated on 45 patients with a mean BMI of 36.7 ± 3.8 kg/m^2^ using the POSE procedure, found no mortalities and only minor complications (low-grade fever, pain, nausea, vomiting, etc.) [11]. In a meta-analysis by Singh et al. assessing a total of 613 patients who underwent POSE, it was found that serious adverse effects were reported in only 2.84 %, and included GI bleeding, extra-gastric bleeding, hepatic abscess, severe pain, severe nausea, and severe vomiting, without listing gastric perforation [14].

In this case report, we encounter a patient who presented one month after a POSE procedure with acute abdomen, symptomized by fever, severe abdominal pain, vomiting, and tachycardia. Imaging revealed that she had pneumoperitoneum and intra-abdominal free fluid secondary to gastric perforation, and she was promptly treated with laparoscopic reversal of POSE and removal of the transmural plications. Although such cases regarding POSE reversal have not been reported in literature, a similar presentation has been encountered post-ESG in a 44-year-old woman, who presented with lower abdominal pain on postoperative day six. In this case, upper gastrointestinal series (UGI) was performed showing large ileus but no evidence of free air, and a subsequent abdominal CT scan revealed large amounts of free air and intraperitoneal fluid. This patient was managed similarly, with removal of sutures until the perforation was identified, followed by closure of the defect [15].

In another study conducted by Alqahtani et al., 1000 patients who underwent ESG were assessed, and only three cases (0.3 %) of ESG reversal were performed, all for reasons other than gastric perforation such as intolerable abdominal pain [12]. Akin to our patient's presentation, four patients (0.4 %) in this study also developed intra-abdominal fluid collection with left-sided pleural effusion (although it is not known whether this was secondary to gastric perforation), however none of these cases were managed with reversal. One was treated conservatively with broad-spectrum antibiotics, while the other three were treated with both image-guided percutaneous drainage and broad-spectrum antibiotics [12].

Lastly, it is important to consider the possibility of this postoperative complication being owed to surgical error or improper technique. As with any surgical procedure, there is a learning curve associated with POSE before proficiency can be achieved. A learning curve is defined as the number of procedures performed that is deemed necessary to reach competency or mastery. This curve is determined by several factors including the operator, operative time, the number of cases performed, complication rate, and the experience of the teaching institution [16]. In a meta-analysis on endoscopic gastric plication procedures by Gys et al., it was found that efficiency for POSE was achieved after a suggested minimal number of 15 procedures [17]. For the sake of illustration, Saumoy et al. found that efficiency for ESG was achieved after an average of 38 procedures, and mastery after 55 [18]. Importantly, a study conducted Sullivan et al. on the safety of endoscopic gastric plication revealed that while extra-gastric bleeding was reported in 1 patient, it was likely due to improper plication placement technique, and that retraining of their surgeons on the proper technique prevented recurrence of such a complication [19]. This highlights that comprehensive training of physicians and ongoing support are critical to ensure optimal procedural outcomes and patient safety, so that each operator reaches a level of proficiency with the equipment and procedure to achieve optimal performance.

## 4 Conclusion

Endoscopic bariatric therapies (EBT) present a promising alternative to bariatric surgery for obesity. While these procedures are an attractive, minimally invasive, and non-surgical weight loss option garnering the interest of many patients who have failed to achieve weight loss via conservative measures, little has been reported on the management of potentially fatal complications associated with these techniques. Typically, patients undergoing EBT face only minor complications (nausea, vomiting, and abdominal pain), which are managed conservatively. Based on our experience with this patient and similar cases in literature, we encourage future studies to further explore the management of such severe complications associated with POSE, particularly indications for reversal, and recommend that more attention be paid to proper training and support for operators of the Incisionless Operating Platform™.

## Availability of data and material

Not applicable.

## Code availability

Not applicable.

## Declaration of competing interest

The authors declare no conflict of interest.
